# Supplementary material for: Novel chemical inhibitor against SOD1 misfolding and aggregation protects neuron-loss and ameliorates disease symptoms in ALS mouse model
Source: Commun Biol. 2021 Dec 15;4:1397. doi: 10.1038/s42003-021-02862-z (PMC8674338; doi:10.1038/s42003-021-02862-z)

Figure S1

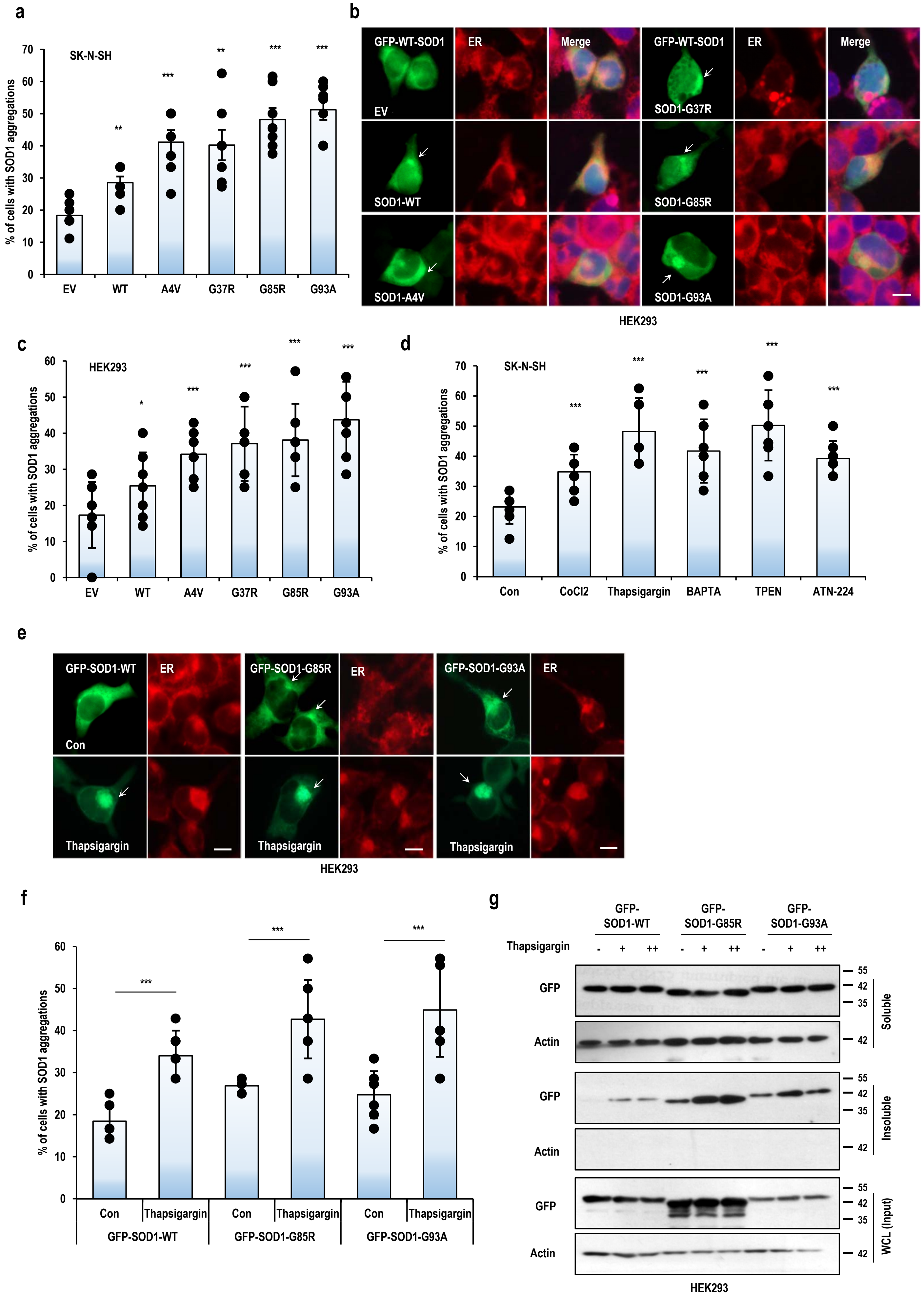
a
SK-N-SH
% of cells with SOD1 aggregations
EV
WT
A4V
G37R
G85R
G93A
b
GFP-WT-SOD1
ER
Merge
EV
SOD1-WT
SOD1-A4V
SOD1-G37R
SOD1-G85R
SOD1-G93A
HEK293
c
HEK293
d
SK-N-SH
Con
CoCl2
Thapsigargin
BAPTA
TPEN
ATN-224
e
GFP-SOD1-WT
GFP-SOD1-G85R
GFP-SOD1-G93A
Con
Thapsigargin
f
GFP-SOD1-WT
GFP-SOD1-G85R
GFP-SOD1-G93A
g
GFP-
SOD1-WT
SOD1-G85R
SOD1-G93A
GFP
Actin
Soluble
Insoluble
WCL (Input)

Fig. S1 The abnormal SOD1 aggregation is induced by MT-SOD1. a-c, Mutant SOD1s induced WT-SOD1 aggregation. **(a)** SK-N-SH cells with SOD1 inclusions (white arrows; strong intensity of SOD1) among GFP positive ones were counted by photomicrographs and the percentages were shown with standard deviation (SD). For cell counting, seven fluorescence images were randomly selected. n = 3 independent experiments; two-tailed Student's t-test. ***P* < 0.01, ****P* < 0.005. **(b)** GFP-WT-SOD1 expressed HEK293 cells were transfected with non-tagged mutant SOD1 (SOD1-A4V, G37R, G85R and G93A) for 24 hr and observed under a fluorescence microscope. Scale bar; 10 µm. **(c)** HEK293 cells with SOD1 inclusions (white arrows; strong intensity of SOD1) among GFP positive ones were counted by photomicrographs and the percentages were shown with standard deviation (SD). For cell counting, seven fluorescence images were randomly selected. n = 3 independent experiments; two-tailed Student's t-test. **P* < 0.05, ****P* < 0.005. **d,** Inclusion positive cells (white arrows; strong intensity of SOD1 in Fig1e) were counted from photomicrographs and the percentages were shown with ± SD. n = 3 independent experiments; two-tailed Student's t-test. ****P* < 0.005. **e-g,** Cellular stresses induced WT- and MT-SOD1 aggregation **(e)** HEK293 cells were transfected with WT- and MT-SOD1 vectors for 24 hr. Thapsigargin (500 nM) was treated for 12 hr. Then, Cells were observed under a fluorescence microscope. Scale bar; 10 µm. **(f)** HEK293 cells with SOD1 inclusions (white arrows; strong intensity of SOD1) among GFP positive ones were counted by photomicrographs and the percentages were shown with standard deviation (SD). For cell counting, seven fluorescence images were randomly selected. n = 3 independent experiments; two-tailed Student's t-test. ****P* < 0.005. **(g)** After transfection with indicating vectors (WT, G85R and G93A-SOD1-GFP) in HEK293 cells for 24 hr and incubated with thapsigargin dose dependently (200, 500 nM). After incubation, the cells were harvested with TNN buffer and centrifuged at 14000 rpm for 30 min then Pellet (insoluble) and supernatant (soluble) were separated.

Figure S2

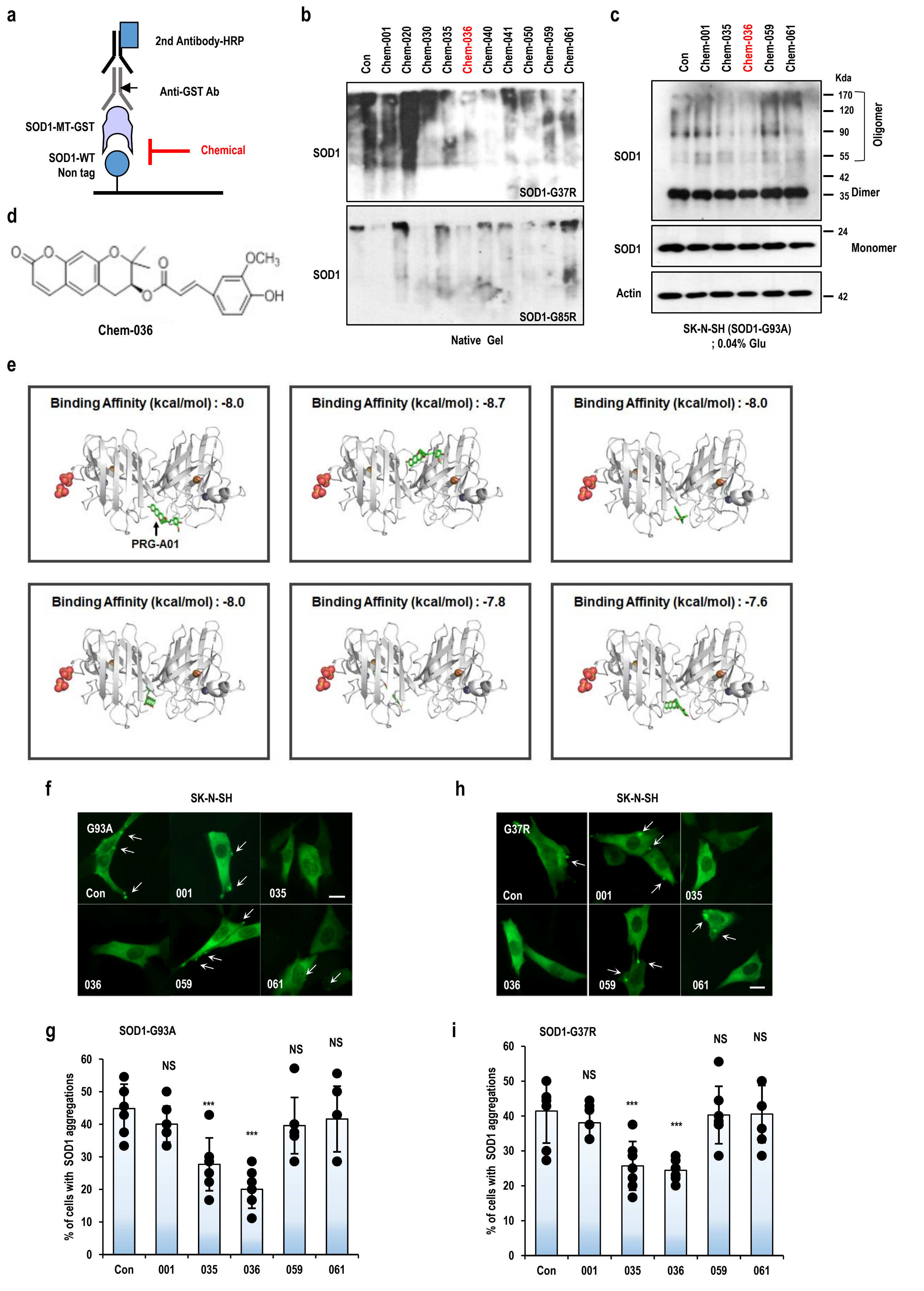
a
2nd Antibody-HRP
Anti-GST Ab
SOD1-MT-GST
Chemical
SOD1-WT
Non tag
b
Con
Chem-001
Chem-020
Chem-030
Chem-035
Chem-036
Chem-040
Chem-041
Chem-050
Chem-059
Chem-061
SOD1
SOD1-G37R
SOD1
SOD1-G85R
Native Gel
c
Con
Chem-001
Chem-035
Chem-036
Chem-059
Chem-061
Kda
170
120
90
55
42
35
Oligomer
Dimer
SOD1
24
SOD1
Monomer
Actin
42
SK-N-SH (SOD1-G93A)
; 0.04% Glu
d
OCH3
OH
Chem-036
e
Binding Affinity (kcal/mol) : -8.0
PRG-A01
Binding Affinity (kcal/mol) : -8.7
Binding Affinity (kcal/mol) : -8.0
Binding Affinity (kcal/mol) : -8.0
Binding Affinity (kcal/mol) : -7.8
Binding Affinity (kcal/mol) : -7.6
f
SK-N-SH
G93A
Con
001
035
036
059
061
h
SK-N-SH
G37R
Con
001
035
036
059
061
g
SOD1-G93A
% of cells with SOD1 aggregations
NS
***
***
NS
NS
Con
001
035
036
059
061
i
SOD1-G37R
% of cells with SOD1 aggregations
NS
***
***
NS
NS
Con
001
035
036
059
061

Figure S2 (Continue)

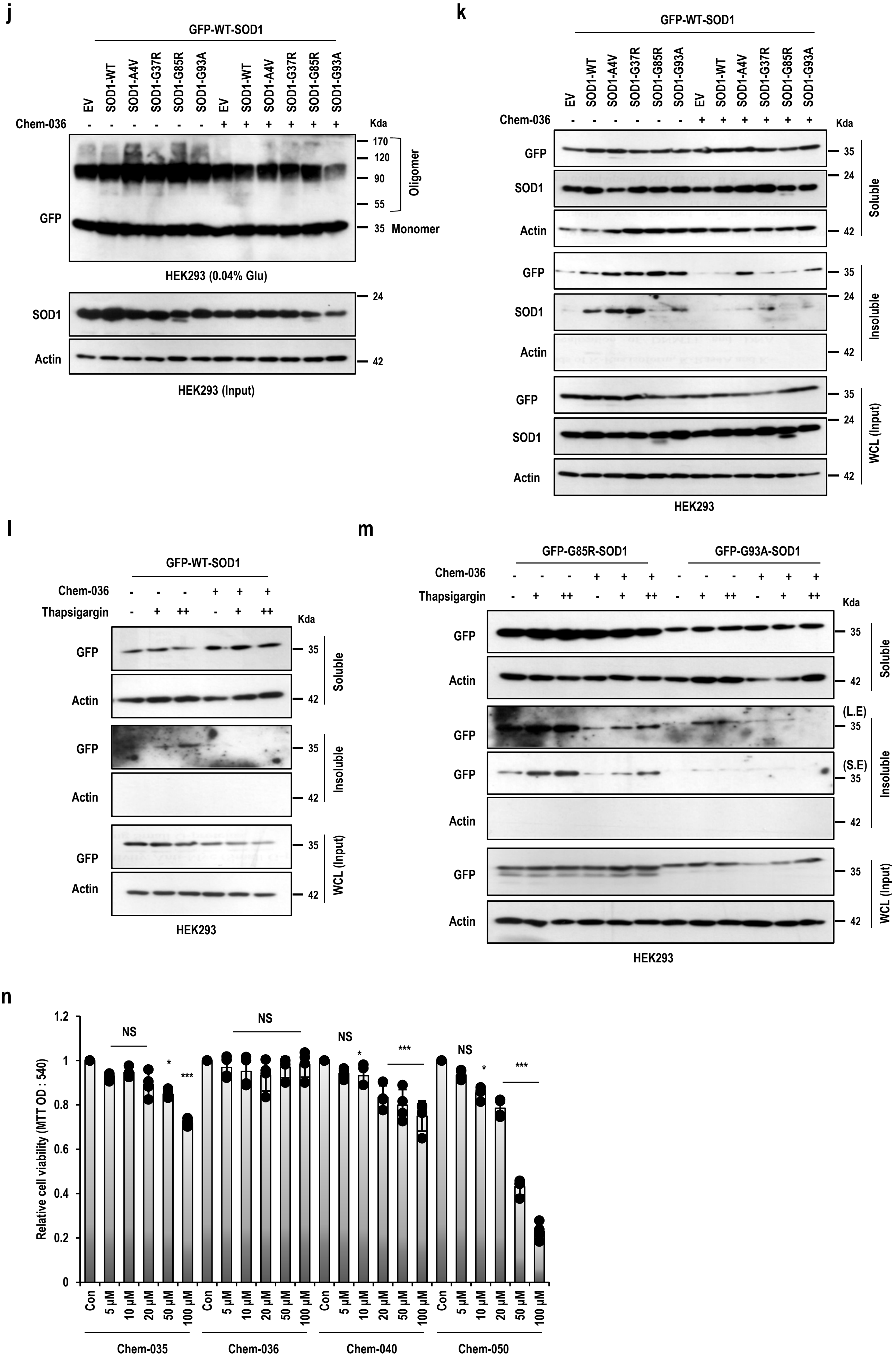

Fig. S2 Chemical screening for SOD1 aggregation inhibitor. a, Diagram for ELISA-based chemical screening system. WT-SOD1 recombinant protein was fixed in a 96-well plate and reacted with each chemical and GST-MT-SOD1 recombinant protein. After 2 hr reaction, GST antibody and HRP-conjugated mouse antibody was incubated. Finally, HRP detection was used with the TMB solution. **b,** Testing the isolated chemicals to check the inhibitory effect on SOD1 aggregation. HEK293 cells were transfected with MT-SOD1 vectors and treated with selected chemicals (5 μM). After incubation, transfected cells were harvested with TNN buffer and separated using native-gel analysis. **c,** Chem-036 reduced oligomerization of MT-SOD1 in SOD1-G93A overexpressed cells. SK-N-SH cells were transfected with SOD1-G93A vectors for 24 hr. After incubation, cells were treated with isolated chemicals (5 μM) for 24 hr and harvested with TNN buffer and reacted with 0.04% glutaraldehyde (Glu) for 1 hr. SDS-PAGE was performed using the SOD1 antibody. Monomer, dimer and oligomer indicated SOD1 formation. **d,** Structure of Chem-036. **e,** The predicted binding sites of Chem-036 on SOD1. Based on PyRx virtual screening program, Chem-036 was bound to the different sites on the surface of the dimeric structure of the native SOD1. **f-i,** Chem-036 suppressed MT-SOD1 inclusions. After transfection with indicating vectors (SOD1-G37R, and G93A) in SK-N-SH cells, chemicals (5 μM) were treated for 24 hr. After incubation, cells were fixed with 4% PFA and SOD1 expression was observed under fluorescence microscope with pan-SOD1 antibody (white arrows; strong intensity of SOD1). Cells with SOD1 inclusions (white arrows; strong intensity of SOD1) were counted by photomicrographs and the percentages were shown with ± SD. n = 3 independent experiments; two-tailed Student's t-test. NS; Not significant, ***P < 0.005. Scale bar; 10 μm. **j,** Chem-036 reduced oligomerization of WT-SOD1 induced with WT and MT-SOD1 overexpression. After co-transfection with indicating vectors (non-tagged WT /MT-SOD1 and GFP-WT-SOD1) in SK-N-SH cells for 24 hr and incubated with chem-036 (5 μM) for 24 hr. Then, cells were harvested with TNNI buffer (50 mM Tris-Cl, pH 7.5, 150 mM NaCl, 0.3% NP-40) and treated with 0.04% glutaraldehyde (Glu) for 1 hr. The samples were performed with SDS-PAGE. Monomer and oligomer indicated SOD1 formation. **k,** Chem-036 reduced the insoluble form of WT-SOD1 induced WT- and MT-SOD1s overexpression. GFP-WT-SOD1 were co-transfected with together non-tagged WT and MT-SOD1 vectors for 24 hr in SK-N-SH cells and treated with Chem-036 (5 μM) for 24 hr. After incubation, the cells were harvested with TNN buffer and centrifuged at 14000 rpm for 30 min then pellet (insoluble) and supernatant (soluble) were separated. Input indicated whole cell lysates (WCL) which were harvested with RIPA buffer. Actin was used as loading control. **l-m,** Chem-036 reduced the insoluble form of WT- MT-SOD1 induced by thapsigargin. HEK293 cells were transfected with GFP-SOD1 (WT, G85R and G93A) expression vector for 24 hr and incubated with thapsigargin (200, 500 nM) and PRG-A01 (5 μM) for 12 hr. After incubation, the cells were harvested with TNN buffer and centrifuged at 14000 rpm for 30 min then pellet (insoluble) and supernatant (soluble) were separated. Input indicated whole cell lysates (WCL) which were harvested with RIPA buffer. Actin was used for loading control. **n,** Chem-036 did not show cytotoxicity. To know the toxic effect of the selected chemical, human normal fibroblast cells were incubated with each chemical for 48 hr dose dependently and measured the viability through MTT assay. "Con" means Dimethyl sulfoxide (DMSO)-treated control. The data are normalized to DMSO-treated cells.n = 3 independent experiments; two-tailed Student's t-test. NS; Not significant, *P < 0.05, ***P < 0.005.

Figure S3

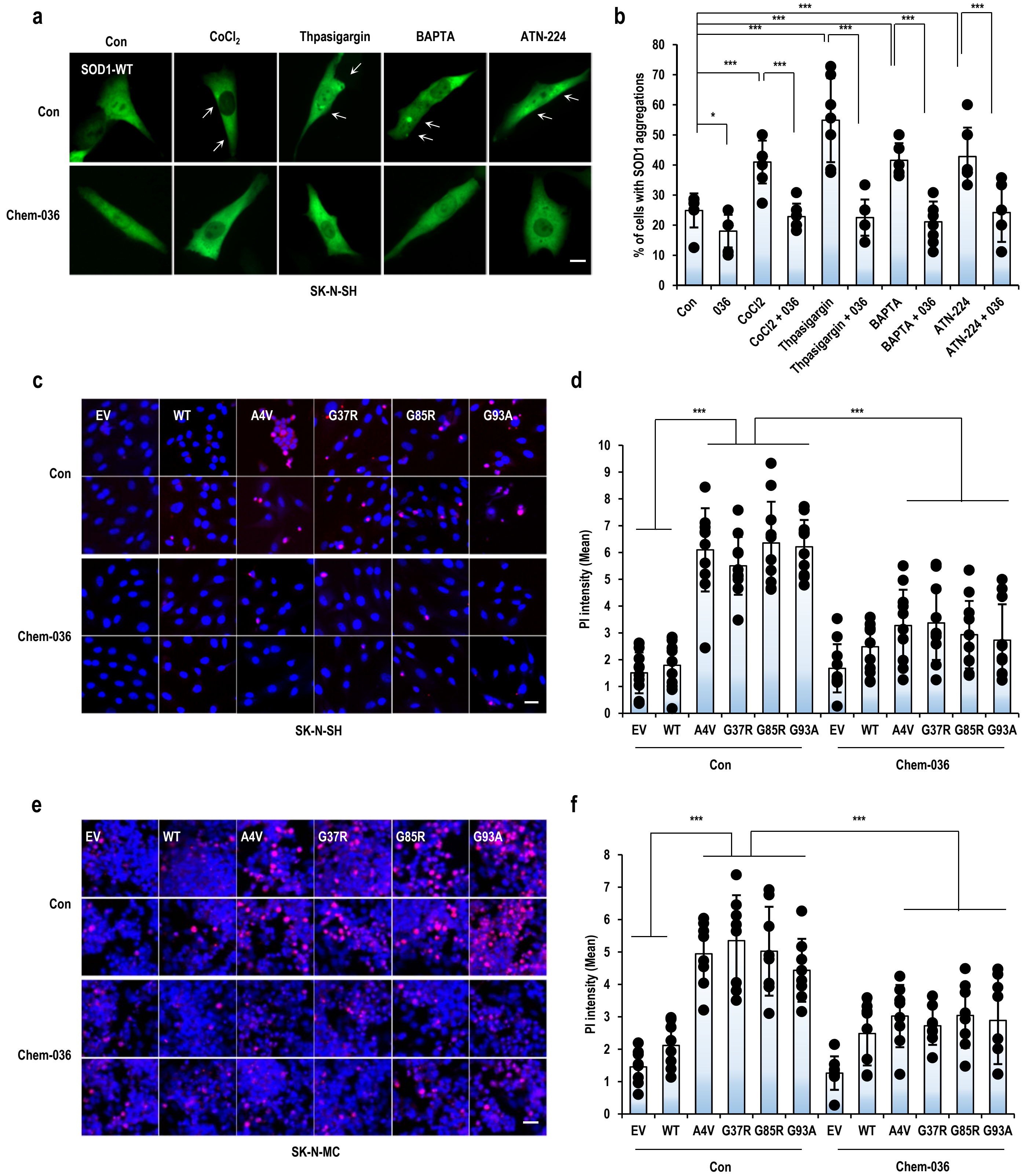

a
Con
CoCl2
Thpasigargin
BAPTA
ATN-224
SOD1-WT
Con
Chem-036
SK-N-SH
b
% of cells with SOD1 aggregations
Con
036
CoCl2
CoCl2 + 036
Thpasigargin
Thpasigargin + 036
BAPTA
BAPTA + 036
ATN-224
ATN-224 + 036
c
EV
WT
A4V
G37R
G85R
G93A
Con
Chem-036
SK-N-SH
d
PI intensity (Mean)
Con
Chem-036
e
EV
WT
A4V
G37R
G85R
G93A
Con
Chem-036
SK-N-MC
f
PI intensity (Mean)
Con
Chem-036

Fig. S3 Chem-036 inhibits SOD1 aggregation and cell death. a-b, Chem-036 reduced cellular stresses-induced WT-SOD1 inclusions. **(a)** Cells were transfected with WT-SOD1 vectors for 24 hr. After incubation, cells were treated with indicated cellular stresses inducer and chem-036 (5 μM) for 12 hr and fixed with 4% PFA. SOD1 expression was observed under fluorescence microscope with pan-SOD1 antibody. Scale bar; 10 μm. **(b)** Cells with SOD1 inclusions (white arrows; strong intensity of SOD1) were counted by photomicrographs and the percentages were shown with ± SD. n = 3 independent experiments; two-tailed Student's t-test. *P < 0.05, ***P < 0.005. **c-f,** Cell death induced by MT-SOD1 overexpression was blocked with chem-036 treatment. After transfection with SOD1 vectors (WT and MT) in SK-N-MC or SK-N-SH cells for 48 hr, PRG-A01 (5 μM) was incubated for 48 hr. We performed propidium iodide (PI; red) staining for detecting dead cells without fixation for 30 min. Then, cells were fixed with 4 % PFA and stained with DAPI (blue) for identifying nucleus of total cells. n = 3 independent experiments; two-tailed Student's t-test. Scale bar; 10 μm. For counting PI intensity, randomly selected fields in images were calculated through the "color histogram" function of the Image J software. ***P < 0.005.

Figure S4

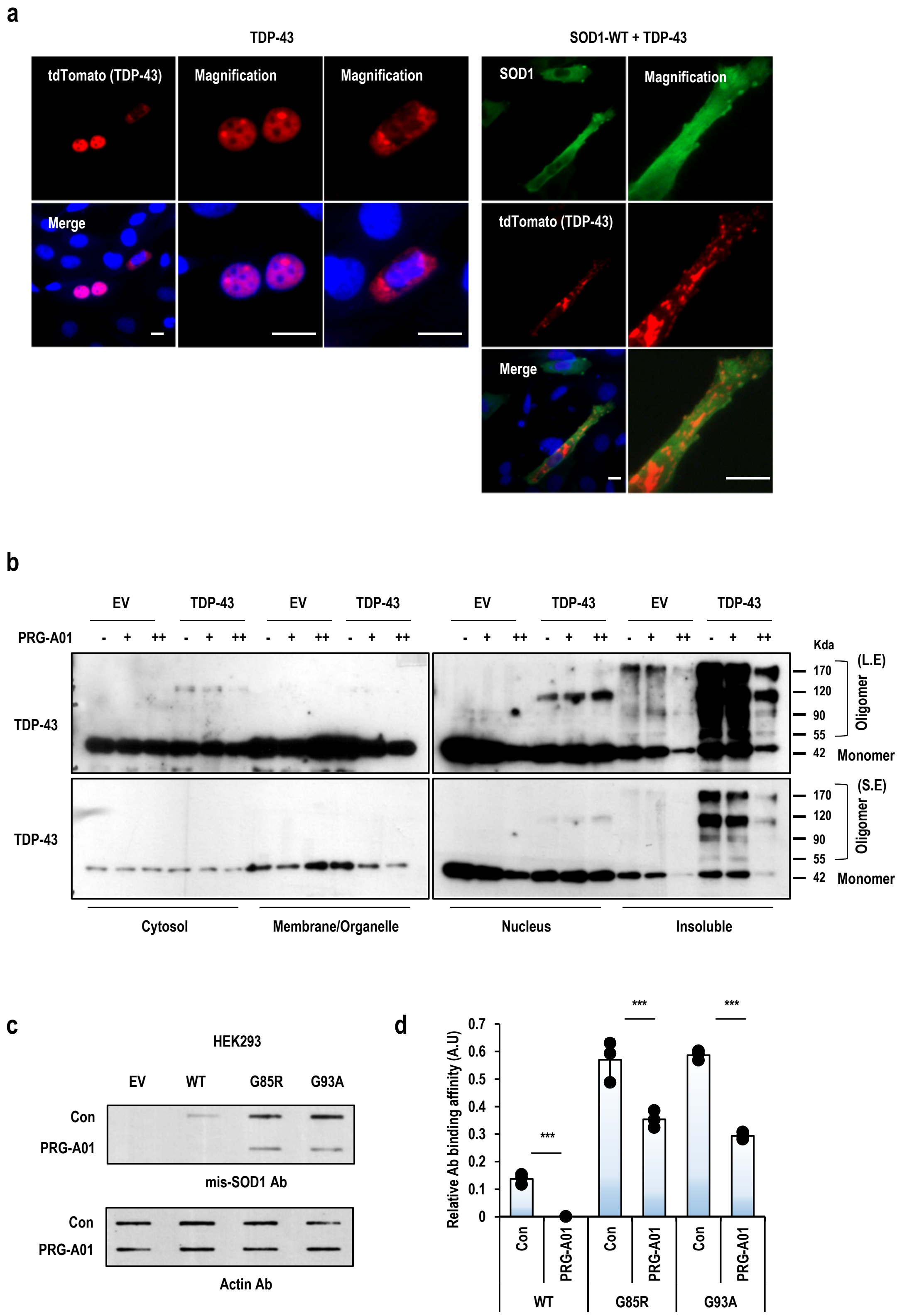
a
TDP-43
SOD1-WT + TDP-43
tdTomato (TDP-43)
Magnification
Magnification
SOD1
Magnification
Merge
tdTomato (TDP-43)
Merge
b
EV
TDP-43
EV
TDP-43
EV
TDP-43
EV
TDP-43
PRG-A01
- + ++ - + ++ - + ++ - + ++ - + ++ - + ++ - + ++ - + ++
Kda
TDP-43
170
120
90
55
42
(L.E)
Oligomer
Monomer
TDP-43
170
120
90
55
42
(S.E)
Oligomer
Monomer
Cytosol
Membrane/Organelle
Nucleus
Insoluble
c
HEK293
EV
WT
G85R
G93A
Con
PRG-A01
mis-SOD1 Ab
Con
PRG-A01
Actin Ab
d
Relative Ab binding affinity (A.U)
0.7
0.6
0.5
0.4
0.3
0.2
0.1
0
***
***
***
Con
PRG-A01
Con
PRG-A01
Con
PRG-A01
WT
G85R
G93A

Fig. S4 PRG-A01 blocks WT-SOD1 aggregation. a, TDP-43 ectopic expression induced WT-SOD1 aggregation. SK-N-SH cells were transfected with WT-SOD1 and tdTomato-TDP-43 for 24 hr. After incubation, cells were fixed with 4% PFA and visualized with IF analysis. Scale bar; 10μm. **b,** Insoluble form of TDP-43 induced by its overexpression reduced with PRG-A01 treatment. Ectopic TDP-43 expression induced cytoplasmic and insoluble TDP-43. SK-N-SH cells were transfected with tdTomato-TDP-43 for 24 hr, then incubated with PRG-A01 dose dependently (2, 5 μM). After incubation, cells were separated into cell fractionation using Subcellular Proteome Extraction Kit. F1, F2, F3 and F4 indicate cytoplasmic (fraction 1), cytoplasmic membrane (fraction 2), nucleus (fraction 3) and insoluble portion (fraction 4). S.E: short exposure, L.E: long exposure. **c,** PRG-A01 reduced misfolding SOD1 induced by MT-SOD1. For detecting of SOD1 conformation, dot blot analysis was performed. HEK293 cells transfected with indicated SOD1 expressing vectors for 24 hr and treated with PRG-A01 (5 μM) for 24 hr. After incubation, cells were lysed with TNN buffer and immobilized on nitrocellulose membrane using dot blot apparatus. The membrane was incubated with misfolding specific SOD1 or Actin antibody. Actin was used as the loading control. **d,** Band density was quantified with Image J software and relative protein expression (mis-SOD1 Ab/Actin Ab) was calculated with ± SD. n = 3 independent experiments; two-tailed Student's t-test. A.U indicate arbitrary units. ***P < 0.005.

Figure S5

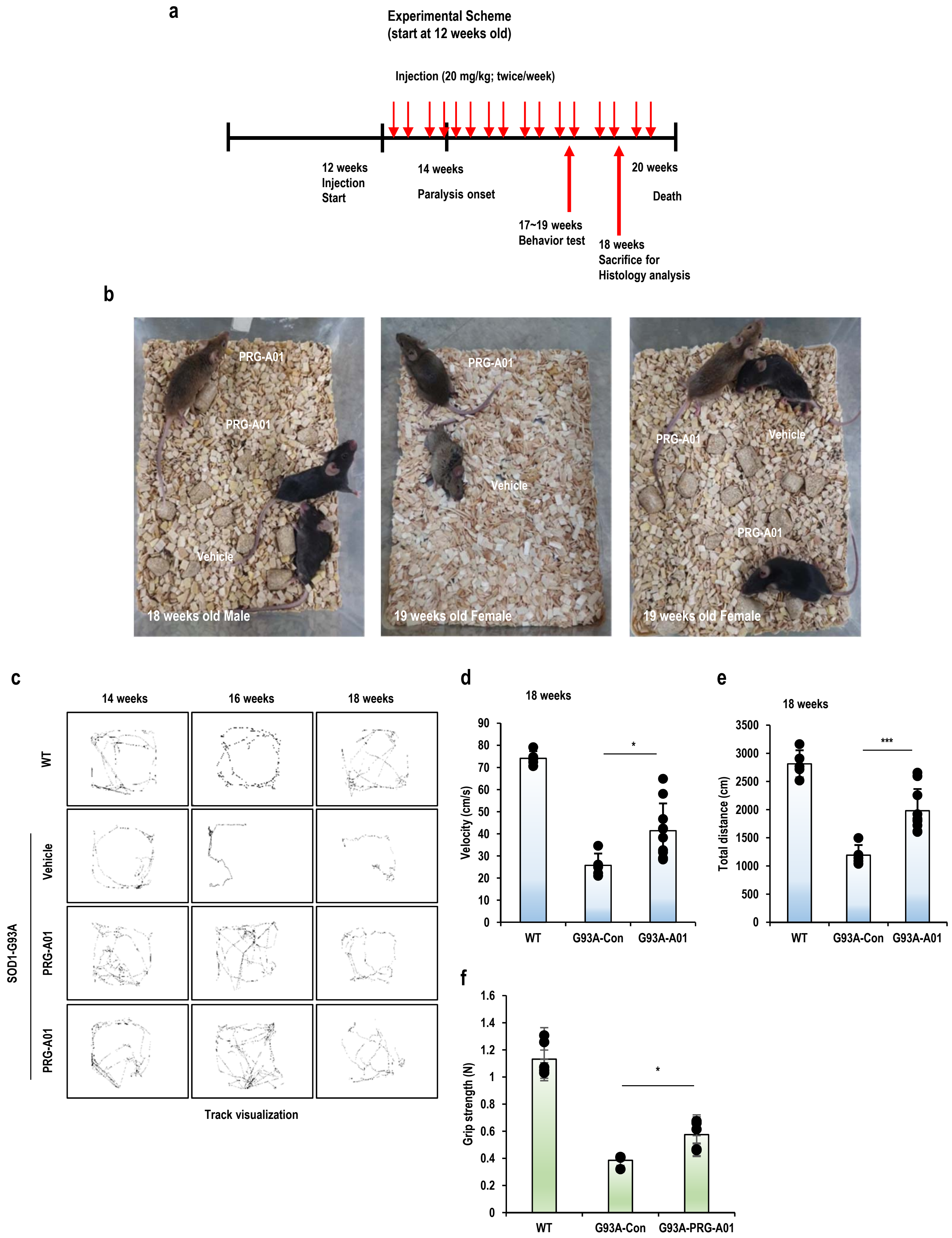
a
Experimental Scheme
(start at 12 weeks old)
Injection (20 mg/kg; twice/week)
12 weeks
Injection
Start
14 weeks
Paralysis onset
20 weeks
Death
17~19 weeks
Behavior test
18 weeks
Sacrifice for
Histology analysis
b
PRG-A01
PRG-A01
Vehicle
18 weeks old Male
PRG-A01
Vehicle
19 weeks old Female
PRG-A01
Vehicle
PRG-A01
19 weeks old Female
c
14 weeks
16 weeks
18 weeks
WT
Vehicle
PRG-A01
PRG-A01
SOD1-G93A
Track visualization
d
18 weeks
Velocity (cm/s)
WT
G93A-Con
G93A-A01
e
18 weeks
Total distance (cm)
WT
G93A-Con
G93A-A01
f
Grip strength (N)
WT
G93A-Con
G93A-PRG-A01

Fig. S5 PRG-A01 ameliorates the mobility regression of SOD1$^{G93A-Tg}$ mouse. a, Experimental scheme for monitoring therapeutic effect of PRG-A01 in SOD1$^{G93A-Tg}$ ALS model mice. 12 weeks old mice were injected with 20 mg/kg, twice per week for 6~8 weeks and recorded their movement at 17-19 weeks. To check the histology analysis, some mouse groups were sacrificed and the spinal cord was isolated at 18 weeks. **b,** Snapshot of mice in the video file. 18 weeks old male (left), 19 weeks old female (middle and right panel) mice were taken the picture. Vehicle-treated mice were lay and did not move. In contrast, PRG-A01 treated mice moved continuously (See supplementary video file 1-5). **c-e,** Open field test of SOD1$^{G93A-Tg}$ ALS model mice with PRG-A01 treatment showed positive effect on moving activity. **(c)** Track visualization analysis was conducted by open field test which results were related with Fig. 4c. **(d)** Velocity **(e)** Total distance of mice carrying SOD1-G93A mutation was recovered in PRG-A01 treated mice. 18 weeks old mice behavior which is shown in Fig.4c and S6 was analyzed with the recorded video files. $*P < 0.05$, $***P < 0.005$. **f,** Measurement of grip strength in the ALS mouse model (chemical was injected from 12 weeks old). Forelimb grip strength was measured at 17 weeks old mice. Comparing to age-matched wild type mouse (n=5), chemical treated mice (n=5) retained about 50% of muscle strength. In contrast, vehicle-treated mice (n=4) showed only 30 % of grip strength. The test was repeated seven times for each mouse. $*P < 0.05$.

Figure S6

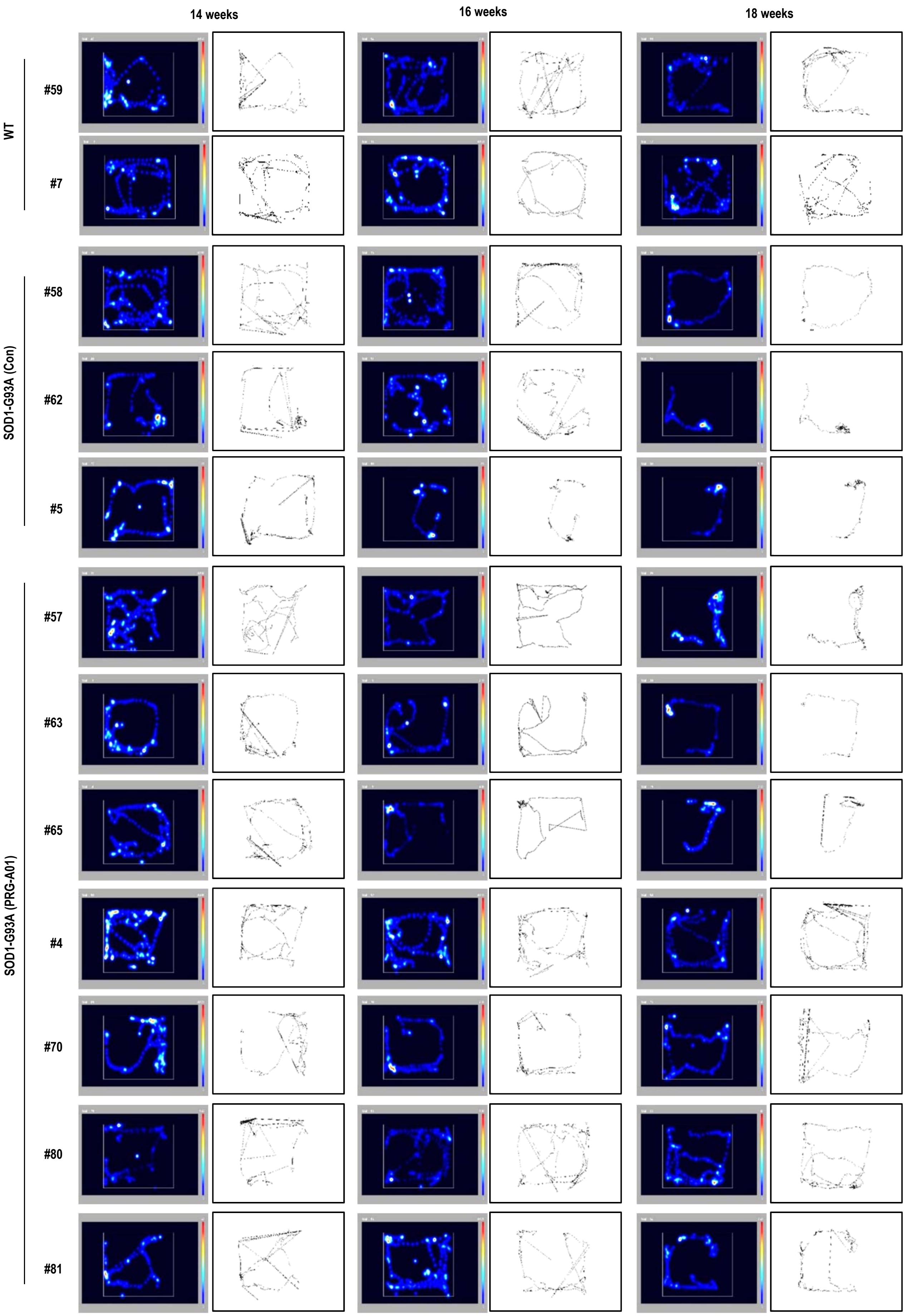

14 weeks
16 weeks
18 weeks
WT
#59
#7
SOD1-G93A (Con)
#58
#62
#5
SOD1-G93A (PRG-A01)
#57
#63
#65
#4
#70
#80
#81

Fig. S6 The activity of SOD1$^{G93A-Tg}$ mouse is maintained in PRG-A01 treated mouse. Comparing to DMSO-treated mice (con, n=4), PRG-A01 treated mice (n=10) maintained the moving activity. SOD1$^{G93A-Tg}$ ALS model mice movements were recorded and performed with open field test. Age-matched WT mice (n=5) were tested as positive control.

Figure S7

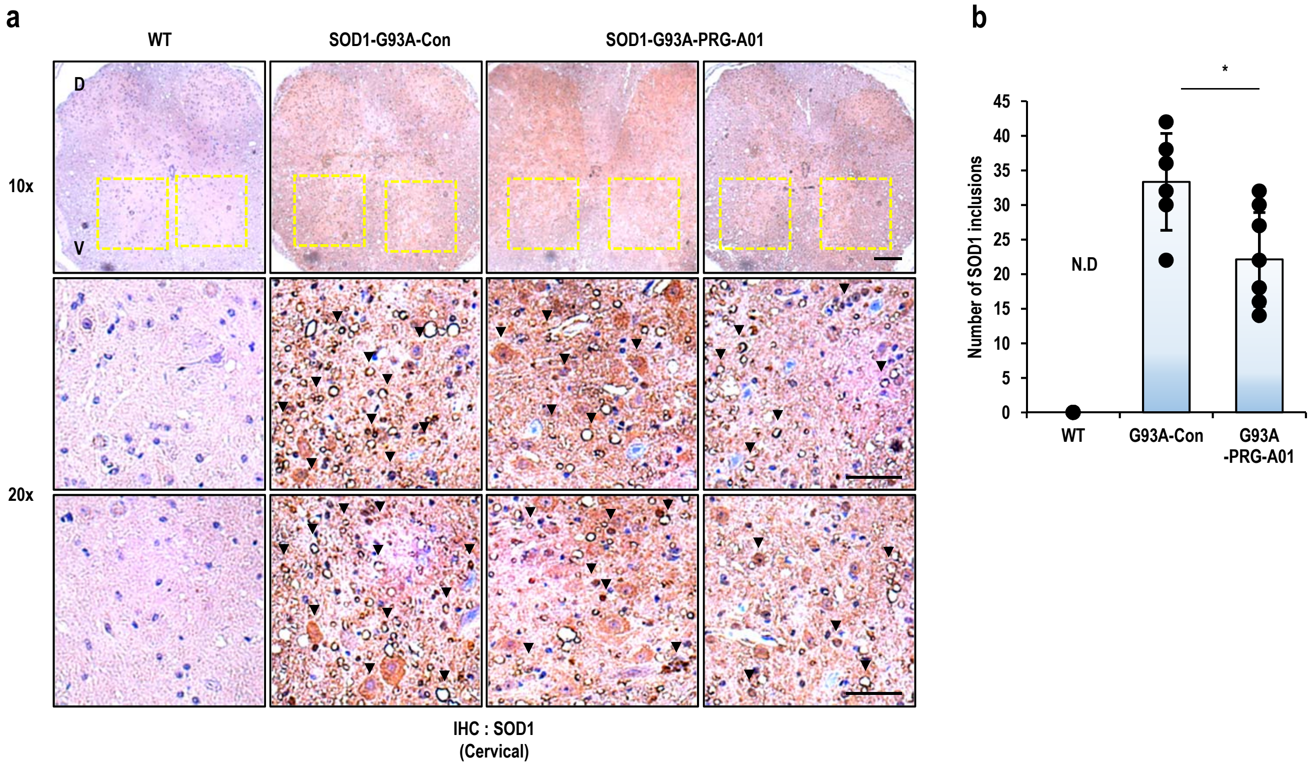

a
WT
SOD1-G93A-Con
SOD1-G93A-PRG-A01
10x
20x
D
V
IHC : SOD1
(Cervical)
b
Number of SOD1 inclusions
0
5
10
15
20
25
30
35
40
45
N.D
*
WT
G93A-Con
G93A
-PRG-A01

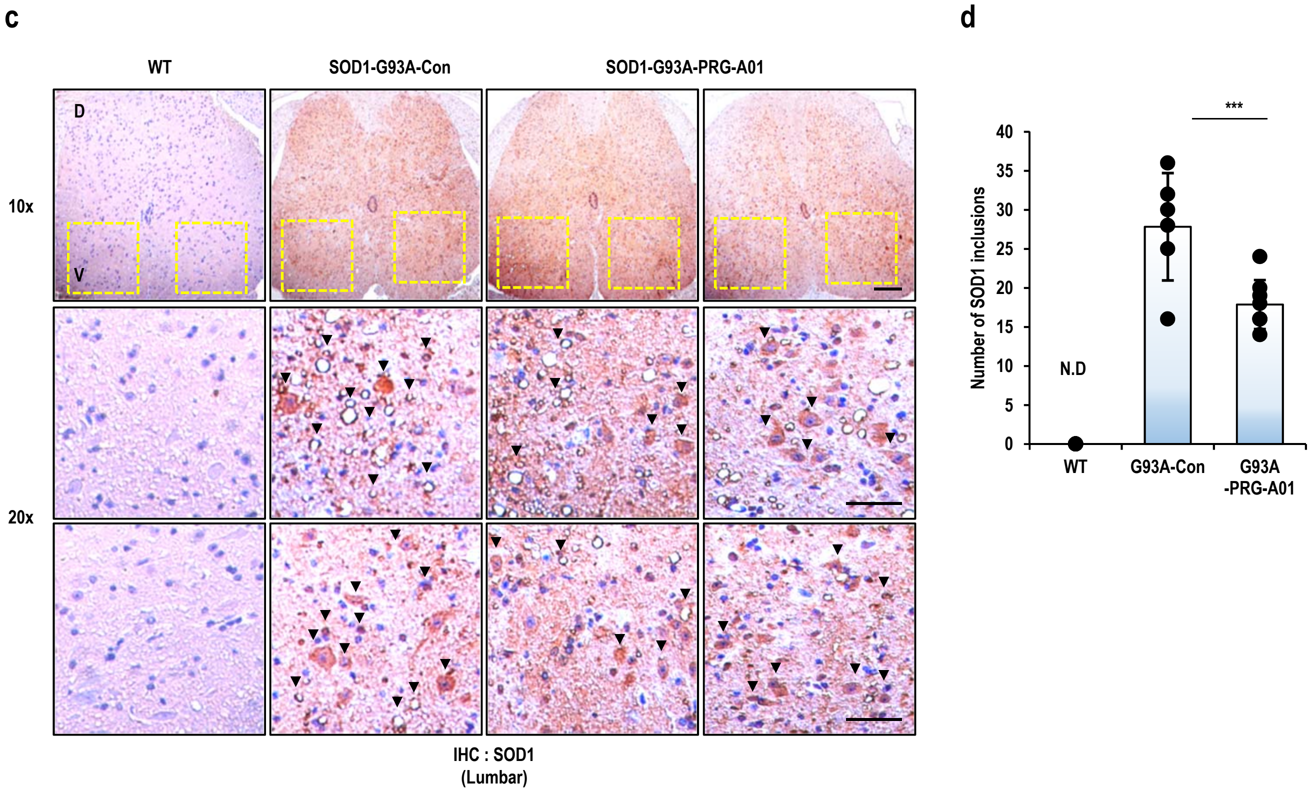

c
WT
SOD1-G93A-Con
SOD1-G93A-PRG-A01
10x
20x
D
V
IHC : SOD1
(Lumbar)
d
Number of SOD1 inclusions
0
5
10
15
20
25
30
35
40
N.D
***
WT
G93A-Con
G93A
-PRG-A01

Fig. S7 PRG-A01 reduces the SOD1 aggregation in SOD1$^{G93A\text{-}Tg}$ spinal cord. a-d, PRG-A01 diminished SOD1-positive vacuoles in the ventral region of the cervical spinal cord **(a-b)** and inclusion of SOD1 in the lumbar spinal cord **(c-d)**. Vehicle (n=3) and PRG-A01 (n=4) injected mouse was sacrificed at 18 weeks then, the spinal cord from mice carrying SOD1-G93A mutation was stained with SOD1 antibody. Scale bar; 20 μm. Representative images were showed with a magnification of 10x and 20x (yellow box). Number of SOD1 inclusions were counted with SOD1-positive vacuoles and plotted in right panel. N.D indicates not detectable. *$P < 0.05$, ***$P < 0.005$.

Figure S8

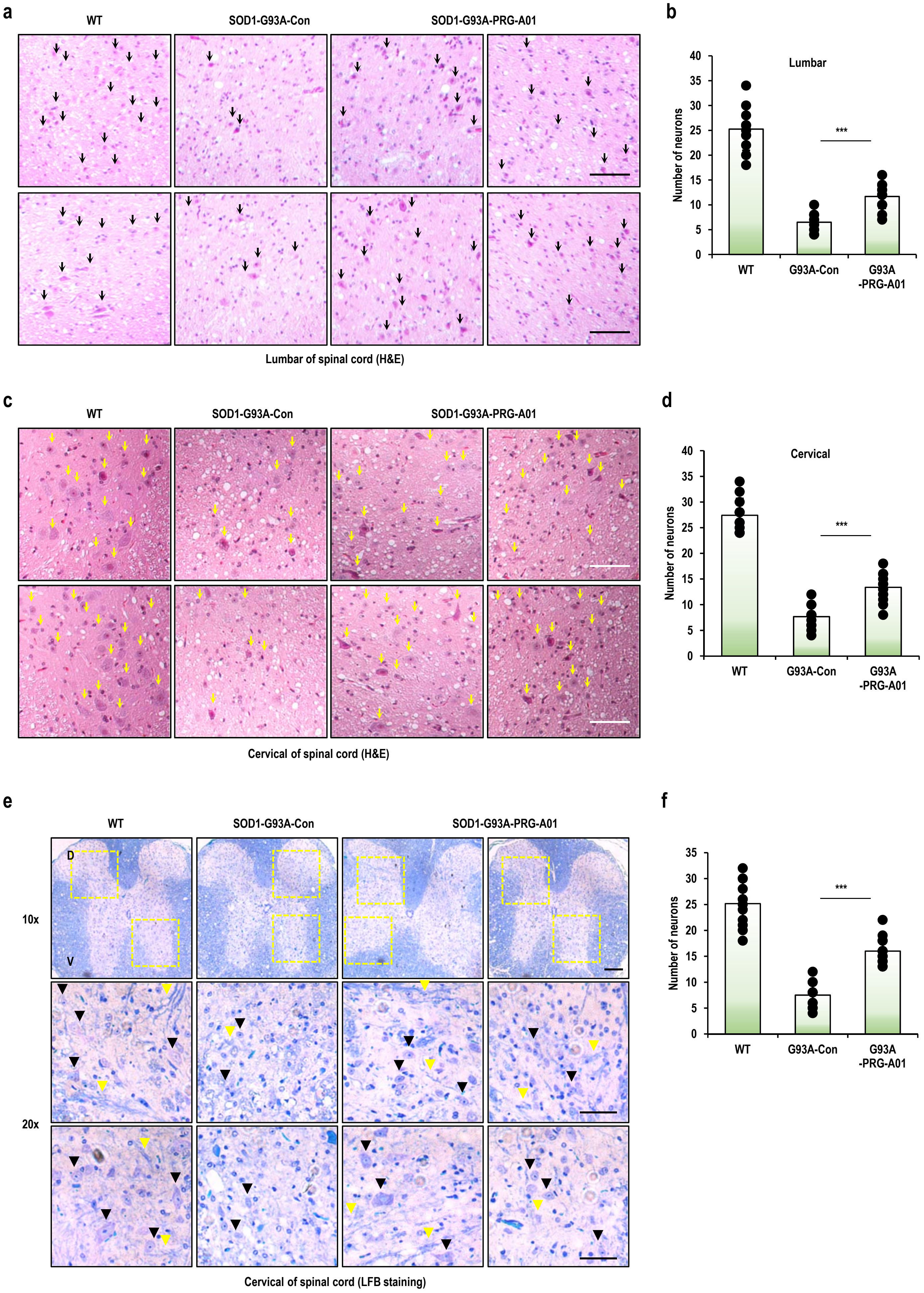
a
WT
SOD1-G93A-Con
SOD1-G93A-PRG-A01
Lumbar of spinal cord (H&E)
b
Lumbar
Number of neurons
40
35
30
25
20
15
10
5
0
***
WT
G93A-Con
G93A
-PRG-A01
c
WT
SOD1-G93A-Con
SOD1-G93A-PRG-A01
Cervical of spinal cord (H&E)
d
Cervical
Number of neurons
40
35
30
25
20
15
10
5
0
***
WT
G93A-Con
G93A
-PRG-A01
e
WT
SOD1-G93A-Con
SOD1-G93A-PRG-A01
D
V
10x
20x
Cervical of spinal cord (LFB staining)
f
Number of neurons
35
30
25
20
15
10
5
0
***
WT
G93A-Con
G93A
-PRG-A01

Fig. S8 Histology analysis of SOD1$^{G93A\text{-}Tg}$ spinal cord. a-d. The 12 weeks old mice were injected with PRG-A01 (20 mg/kg) for 6 weeks and sacrificed at 18 weeks. The lumbar spinal cord **(a-b)** and cervical spinal cord **(c-d)** was stained with H&E. Large amount of spinal nerve cells (black and yellow arrowheads) was maintained in PRG-A01 treatment mice (n=4), compared to vehicle-treated mice (n=3). Scale bar; 20 μm. Representative images were showed and number of neurons were counted and plotted in right panel. ***$P < 0.005$. **e-f.** PRG-A01 ameliorated demyelination in the gray matter of the cervical spinal cord. A considerable amount of nerve cells (black arrowheads) and myelin (yellow arrowheads) was showed in PRG-A01 treated mice (n=4). The cervical spinal cord was stained with Luxol fast blue (LFB) staining. Representative images were showed with a magnification of 10x and 20x (yellow box). Boxes indicate magnified regions displayed in the bottom panel. Scale bar; 20 μm. Number of neurons were counted and plotted in right panel. ***$P < 0.005$.

Figure S9

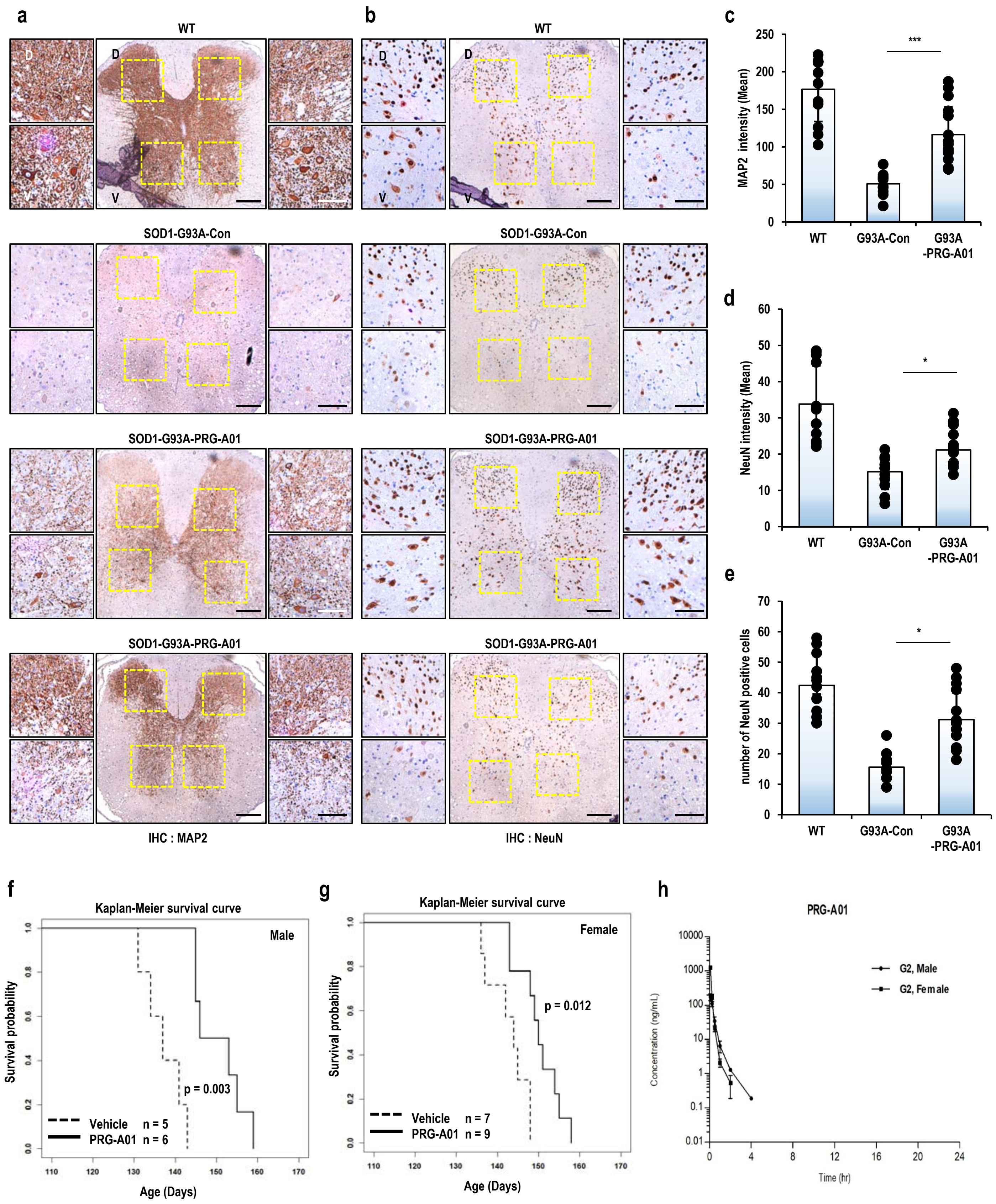

a
b
c
d
e
f
g
h
WT
D
V
SOD1-G93A-Con
SOD1-G93A-PRG-A01
IHC : MAP2
IHC : NeuN
MAP2 intensity (Mean)
***
WT
G93A-Con
G93A -PRG-A01
NeuN intensity (Mean)
*
number of NeuN positive cells
Kaplan-Meier survival curve
Male
Female
p = 0.003
p = 0.012
Vehicle n = 5
PRG-A01 n = 6
Vehicle n = 7
PRG-A01 n = 9
Survival probability
Age (Days)
PRG-A01
G2, Male
G2, Female
Concentration (ng/mL)
Time (hr)

Fig. S9 PRG-A01 conserves the neuronal markers in SOD1$^{G93A\text{-}Tg}$ spinal cord. a-b, PRG-A01 ameliorated the reduction of MAP2 **(a)** and NeuN **(b)** intensity in the cervical spinal cord of SOD1$^{G93A\text{-}Tg}$ mice. The neuronal markers were maintained in PRG-A01 injected mice (n=4) in dorsal and ventral horn, compared to vehicle-treated mice (n=3), Scale bar; 20 μm. Representative images were showed with a magnification of 10x and 20x (yellow box). Boxes indicate magnified regions displayed in the left or right panel. **c-d,** The intensity of neuronal markers (MAP2 and NeuN) was counted with Image j software. For counting intensity, IHC images were divided using the "color deconvolution" function in Image J software and quantified the DAB signal. **e,** Number of NeuN positive cells were counted in ventral and dorsal horn of the spinal cord. $^*P < 0.05$, $^{***}P < 0.005$. **f-g,** Kaplan Meier survival curve of SOD1$^{G93A\text{-}Tg}$ ALS model mice. Comparing to DMSO-treated mice (Con, n=12), PRG-A01-treated mice (n=15) showed extended life span about 13 days (Male) and 8 days (Female) respectively. $^{***}P < 0.005$. **h,** Pharmacokinetic analysis of PRG-A01. Due to the extremely rapid degradation of PRG-A01 in blood as well as gastro-intestinal tract, we cannot obtain the bioavailability (BA).

Fig. 1b

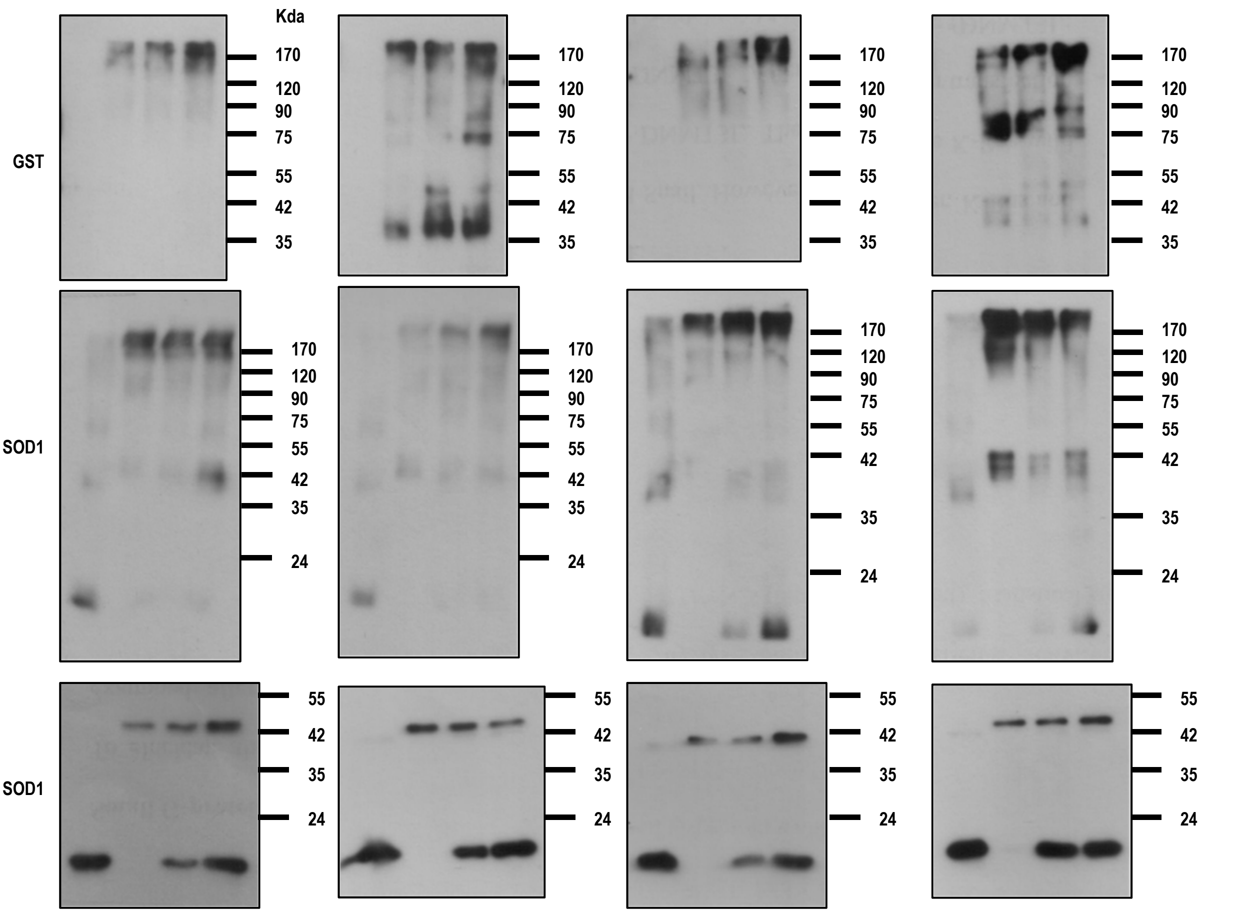

Fig. 1c

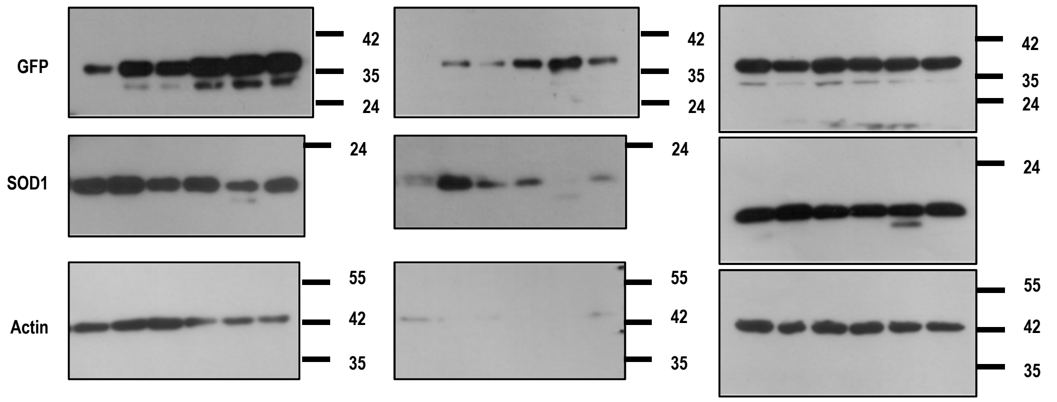

Fig. 1d

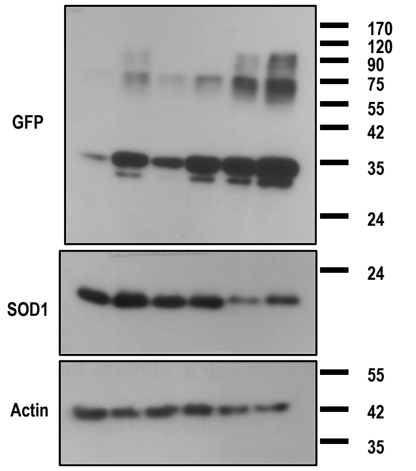

Fig. 1f

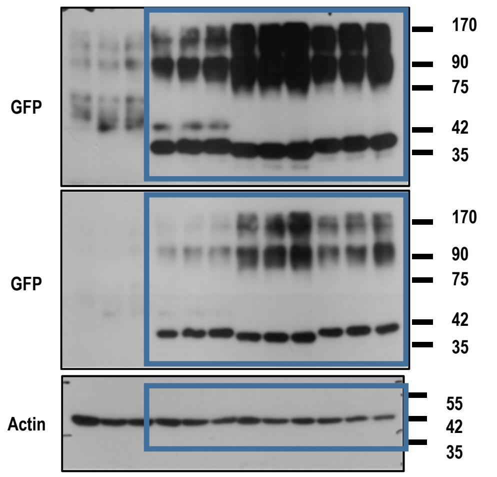

Fig. 1g

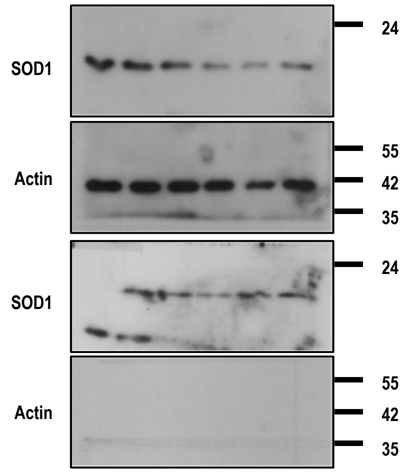

Fig. 2b

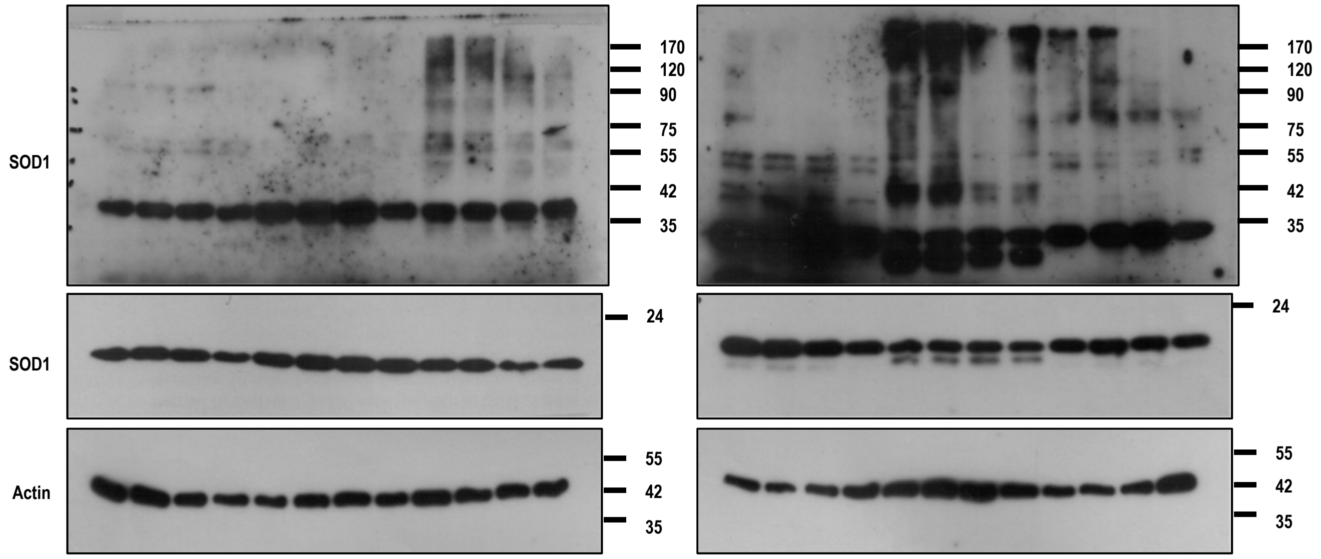

Fig. 2e

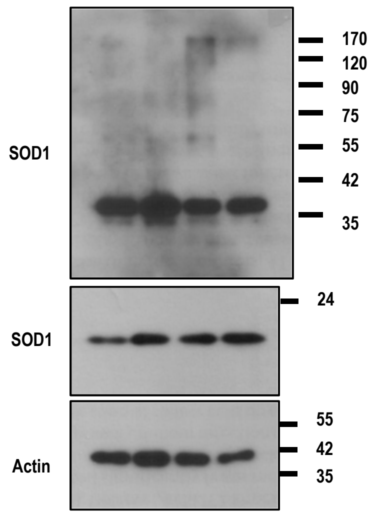

Fig. 3e

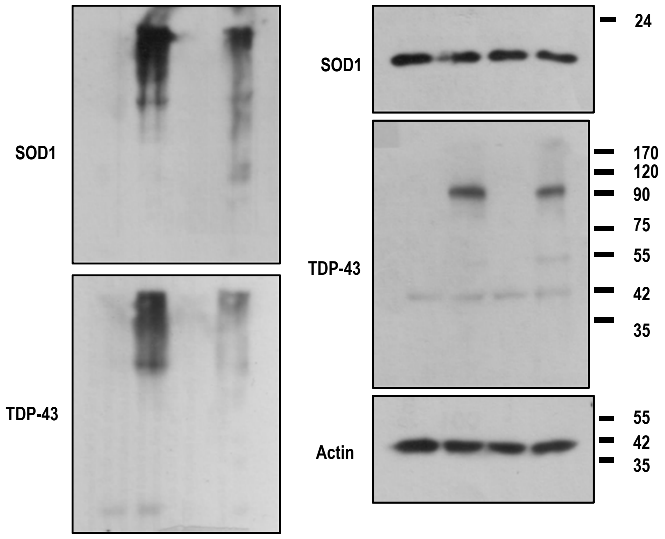

Fig. 3f

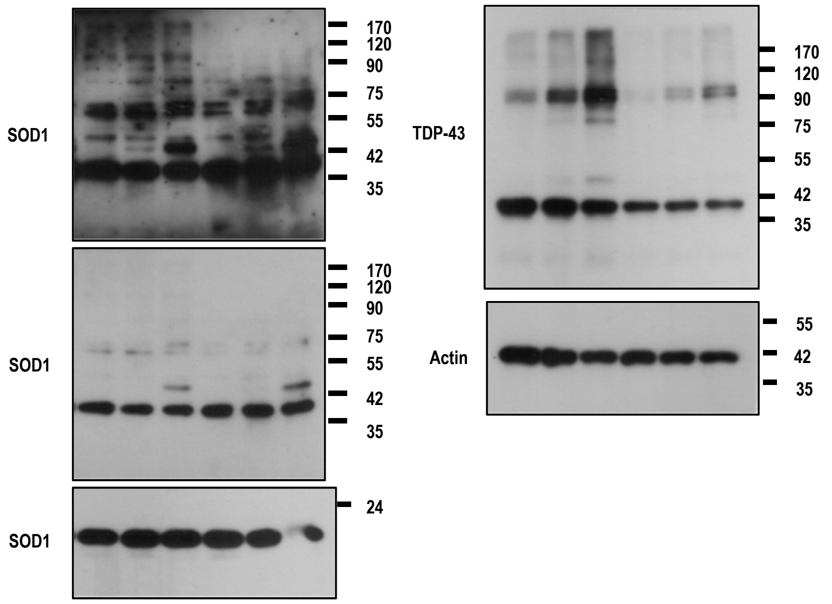

Fig. 3g

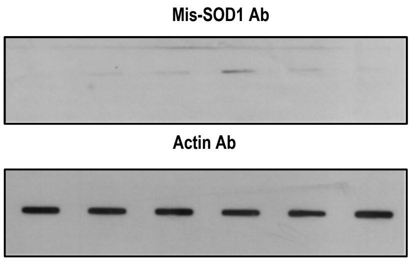

Fig. 3i

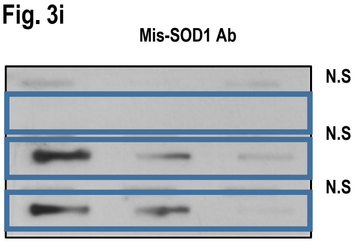

Fig. S1g

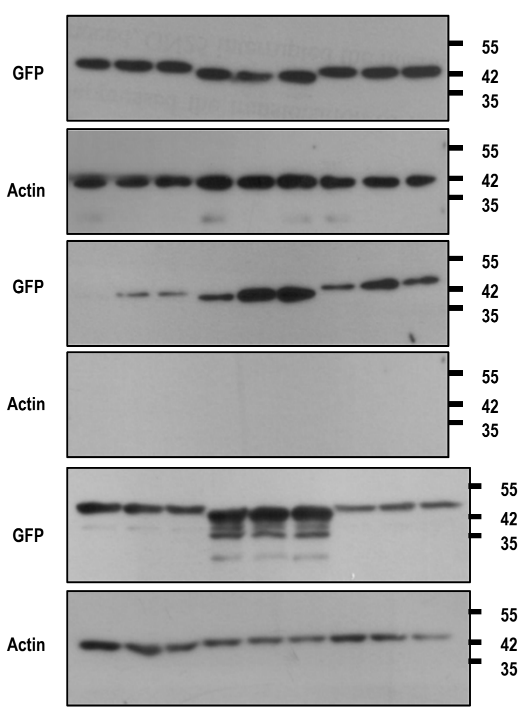

Fig. S2b

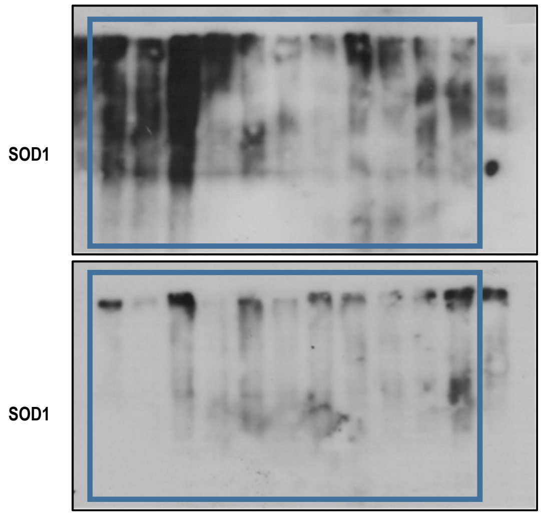

Fig. S2c

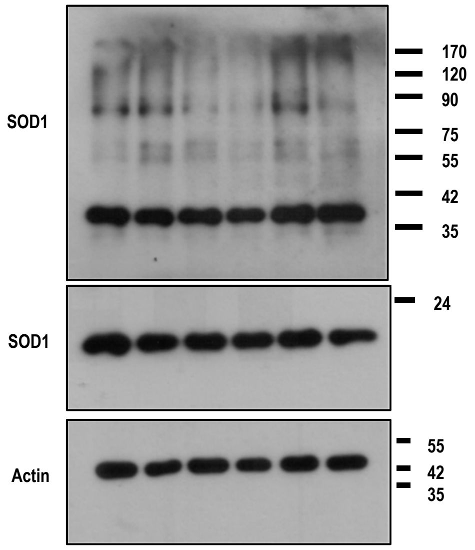

Fig. S2j

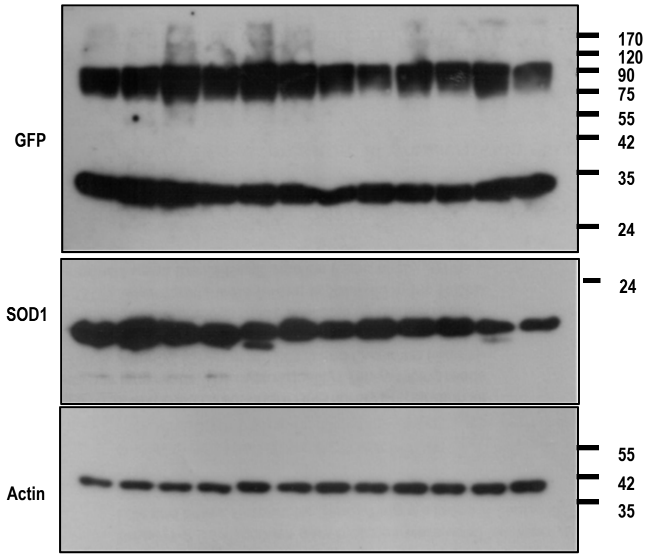

Fig. S2k

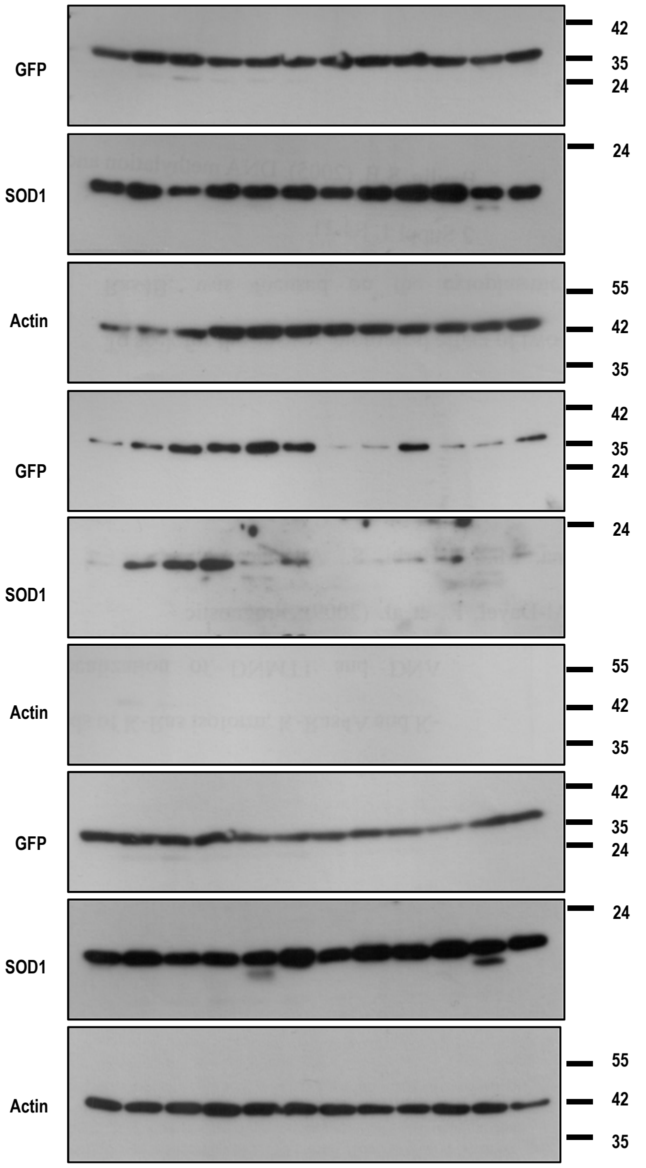

Fig. S2l

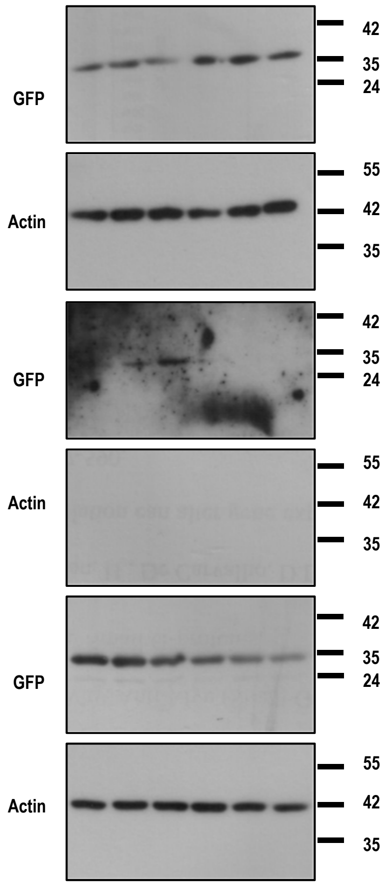

Fig. S2m

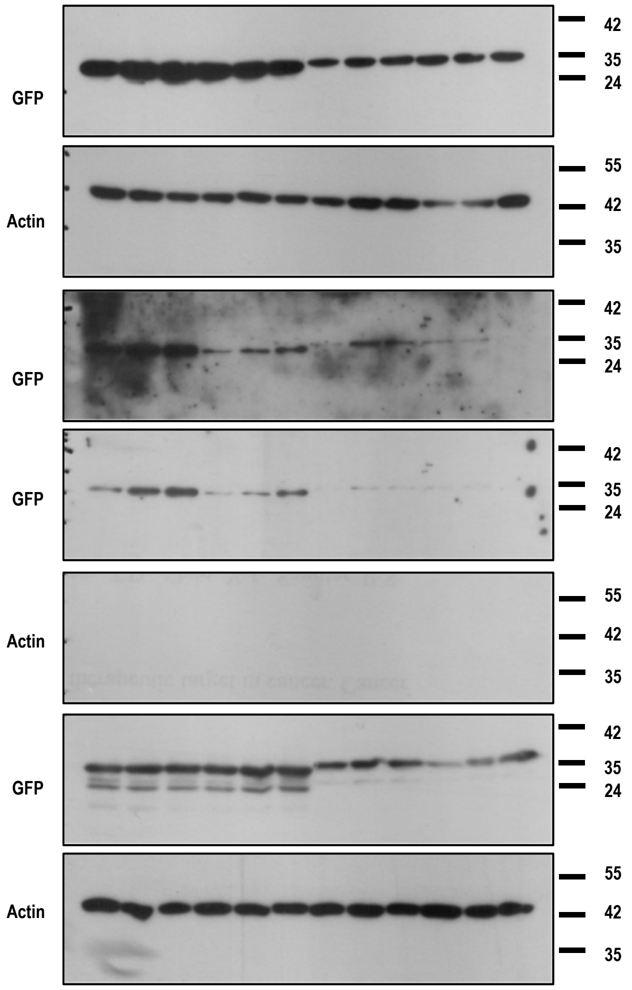

Fig. S4b

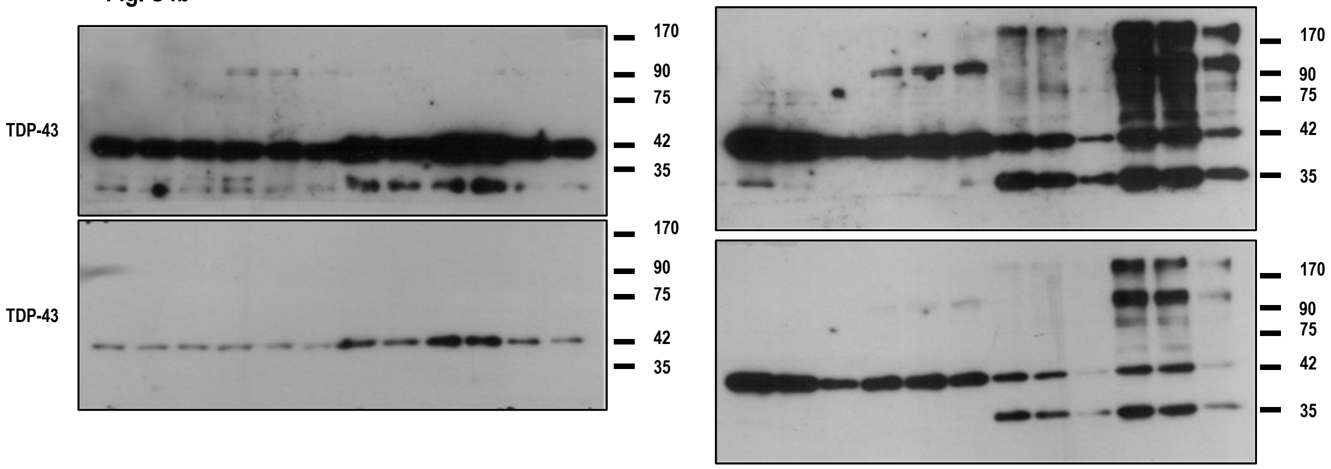

Fig. S4c

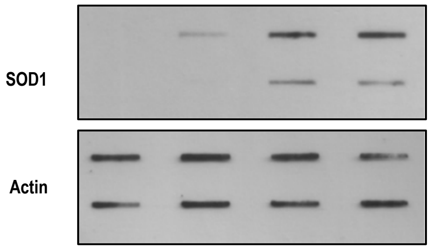

Supplement: Supplementary file 2 — Supplementary Information [file 42003_2021_2862_MOESM2_ESM.pdf]
