# Supplementary material for: Novel chemical inhibitor against SOD1 misfolding and aggregation protects neuron-loss and ameliorates disease symptoms in ALS mouse model
Source: Commun Biol. 2021 Dec 15;4:1397. doi: 10.1038/s42003-021-02862-z (PMC8674338; doi:10.1038/s42003-021-02862-z)
Supplement: Supplementary file 3 — Description of Additional Supplementary Files [file 42003_2021_2862_MOESM3_ESM.pdf]

Description of Additional Supplementary Files

File name: Supplementary Movie 1
Description: Movie of 18 weeks old WT male mouse**.**

File name: Supplementary Movie 2
Description: Movie of 18 weeks old SOD1 G93A-Tg male mice. Comparing to vehicle-treated mice (upper-right mouse), which lay and cannot move, two injected mice are continuously moving in the cage.

File name: Supplementary Movie 3
Description: Movie of 19 weeks old WT female mouse.

File name: Supplementary Movie 4
Description: Movie of 19 weeks old SOD1G93A-Tg female mice. Vehicle-treated mouse (center) cannot move anymore and shows the difficulty in breathing. In contrast, PRG-A01 treated mouse still show the movement ability, although mice have some problem in hind leg movement.

File name: Supplementary Movie 5
Description: Movie of 19 weeks old SOD1G93A-Tg female mice. Vehicle-treated mouse (upper-left) shows complete paralysis of hind legs and shows the difficulty in standing. In contrast, the other two mice (PRG-A01 treated) are moving continuously.

File name: Supplementary Data 1
Description:

Fig 2d. Percent of cells with SOD1 aggregations after treatment with chemicals in SK-N-SH

Fig 2g. Percent of cells with SOD1 aggregations after treatment with Chem-036 and TPEN in SK-N-SH

Fig 3b. Cytoplasm/Nucleus ratio of TDP-43 localization after treatment with PRG-A01 in SK-N-SH

Fig 3c. Percent of cells with TDP-43 cytoplasmic inclusions after treatment with PRG-A01 in SK-N-SH

Fig 3d. Percent of cells with WT-SOD1 aggregations after treatment with PRG-A01 in SK-N-SH

Fig 3h. Intensity of Dot blot analysis in SK-N-SH (A.U)

Fig 4b. Body weight after I.P injection with PRG-A01 in SOD1G93A-Tg mice

Fig 4d. Velocity after I.P injection with PRG-A01 in SOD1G93A-Tg mice

Fig 4e. Distance after I.P injection with PRG-A01 in SOD1G93A-Tg mice

Fig 4g. Number of SOD1 inclusions after I.P injection with PRG-A01 in cervical spinal cord of SOD1G93ATg mice

Fig S1a. Percent of cells with SOD1 aggregations in SK-N-SH

Fig S1c. Percent of cells with SOD1 aggregations in HEK293

Fig S1d. Percent of cells with SOD1 aggregations after treatment with cellular stresses in SK-N-SH

Fig S1f. Percent of cells with SOD1 aggregations after treatment with cellular stresses in HEK293

Fig S2g. Percent of cells with SOD1 aggregations after treatment with chemicals in SK-N-SH overexpressed SOD1-G93A

Fig S2i. Percent of cells with SOD1 aggregations after treatment with chemicals in SK-N-SH overexpressed SOD1-G37R

Fig S2n. Relative cell viability after treatment with chemicals in normal fibroblast

Fig S3b. Percent of cells with SOD1 aggregations after treatment with cellular stresses and Chem-036 in SK-N-SH

Fig S3d. PI intensity (mean) after treatment with Chem-036 in SK-N-SH

Fig S3f. PI intensity (mean) after treatment with Chem-036 in SK-N-MC

Fig S4d. Intensity of Dot blot analysis in HEK293 (A.U)

Fig S5d. Velocity at 18weeks after I.P injection with PRG-A01 in SOD1G93A-Tg mice

Fig S5e. Distance at 18weeks after I.P injection with PRG-A01 in SOD1G93A-Tg mice

Fig S5f. Grip strength (N)

Fig S7b. Number of SOD1 inclusions after I.P injection with PRG-A01 in cervical spinal cord of SOD1G93ATg mice

Fig S7d. Number of SOD1 inclusions after I.P injection with PRG-A01 in lumbar spinal cord of SOD1G93ATg mice

Fig S8b. Number of neurons after I.P injection with PRG-A01 in lumbar spinal cord of SOD1G93A-Tg mice (H&E)

Fig S8d. Number of neurons after I.P injection with PRG-A01 in cervical spinal cord of SOD1G93A-Tg mice (H&E)

Fig S8f. Number of neurons after I.P injection with PRG-A01 in cervical spinal cord of SOD1G93A-Tg mice (LFB)

Fig S9c. MAP2 intensity (mean) after I.P injection with PRG-A01 in spinal cord of SOD1G93A-Tg mice

Fig S9d. NeuN intensity (mean) after I.P injection with PRG-A01 in spinal cord of SOD1G93A-Tg mice

Fig S9e. Number of NeuN positive cells after I.P injection with PRG-A01 in spinal cord of SOD1G93A-Tg mice
