# Supplementary material for: Novel chemical inhibitor against SOD1 misfolding and aggregation protects neuron-loss and ameliorates disease symptoms in ALS mouse model
Source: Commun Biol. 2021 Dec 15;4:1397. doi: 10.1038/s42003-021-02862-z (PMC8674338; doi:10.1038/s42003-021-02862-z)
Supplement: Supplementary file 10 — Reporting Summary [file 42003_2021_2862_MOESM10_ESM.pdf]

nature portfolio

Corresponding author(s): Double-blind peer review submissions : COMMSBIO-21-1471-A

Last updated by author(s): Oct 17, 2021

Reporting Summary

Nature Portfolio wishes to improve the reproducibility of the work that we publish. This form provides structure for consistency and transparency in reporting. For further information on Nature Portfolio policies, see our Editorial Policies and the Editorial Policy Checklist.

Statistics

For all statistical analyses, confirm that the following items are present in the figure legend, table legend, main text, or Methods section.

n/a	Confirmed	
☐	☒	The exact sample size (n) for each experimental group/condition, given as a discrete number and unit of measurement
☐	☒	A statement on whether measurements were taken from distinct samples or whether the same sample was measured repeatedly
☐	☒	The statistical test(s) used AND whether they are one- or two-sided<br>*Only common tests should be described solely by name; describe more complex techniques in the Methods section.*
☒	☐	A description of all covariates tested
☐	☒	A description of any assumptions or corrections, such as tests of normality and adjustment for multiple comparisons
☐	☒	A full description of the statistical parameters including central tendency (e.g. means) or other basic estimates (e.g. regression coefficient) AND variation (e.g. standard deviation) or associated estimates of uncertainty (e.g. confidence intervals)
☐	☒	For null hypothesis testing, the test statistic (e.g. F, t, r) with confidence intervals, effect sizes, degrees of freedom and P value noted<br>*Give P values as exact values whenever suitable.*
☒	☐	For Bayesian analysis, information on the choice of priors and Markov chain Monte Carlo settings
☒	☐	For hierarchical and complex designs, identification of the appropriate level for tests and full reporting of outcomes
☒	☐	Estimates of effect sizes (e.g. Cohen's d, Pearson's r), indicating how they were calculated

Our web collection on statistics for biologists contains articles on many of the points above.

Software and code

Policy information about availability of computer code

Data collection	No software was used
Data analysis	Image J was used for quantification of WB, IF images, histology and IHC image analysis<br>Microsoft Excel (version 2016) was used for statistics data analysis<br>EthoVision XT 15 software (Noldus) was used for analyzing recorded mouse movement video file

For manuscripts utilizing custom algorithms or software that are central to the research but not yet described in published literature, software must be made available to editors and reviewers. We strongly encourage code deposition in a community repository (e.g. GitHub). See the Nature Portfolio guidelines for submitting code & software for further information.

Data

Policy information about availability of data

All manuscripts must include a data availability statement. This statement should provide the following information, where applicable:

- Accession codes, unique identifiers, or web links for publicly available datasets
- A description of any restrictions on data availability
- For clinical datasets or third party data, please ensure that the statement adheres to our policy

The datasets generated during and/or analysed during the current study are available from the corresponding author on reasonable request

Field-specific reporting

Please select the one below that is the best fit for your research. If you are not sure, read the appropriate sections before making your selection.

☒ Life sciences ☐ Behavioural & social sciences ☐ Ecological, evolutionary & environmental sciences

For a reference copy of the document with all sections, see nature.com/documents/nr-reporting-summary-flat.pdf

Life sciences study design

All studies must disclose on these points even when the disclosure is negative.

Sample size	Sample size was determined based on previous reports or similar studies in this field. Sample size for in vivo experiments were determined as reasonable number of animals in which statistically significant differences were observed between groups.
Data exclusions	No data was excluded.
Replication	All replication attempt were successful in western blot, IF, and in vivo experiments. All For final quantification, all samples were quantified for a minimum of three samples.
Randomization	All experiments were performed with randomization when necessary. Mice were randomly grouped with age, gender-matched littermates prior to injection. Cell counting images were selected randomly
Blinding	All data analysis were performed in a blinded manner.

Reporting for specific materials, systems and methods

We require information from authors about some types of materials, experimental systems and methods used in many studies. Here, indicate whether each material, system or method listed is relevant to your study. If you are not sure if a list item applies to your research, read the appropriate section before selecting a response.

Materials & experimental systems

n/a	Involved in the study	
☐	☒	Antibodies
☐	☒	Eukaryotic cell lines
☒	☐	Palaeontology and archaeology
☐	☒	Animals and other organisms
☒	☐	Human research participants
☒	☐	Clinical data
☒	☐	Dual use research of concern

Methods

n/a	Involved in the study	
☒	☐	ChIP-seq
☒	☐	Flow cytometry
☒	☐	MRI-based neuroimaging

Antibodies

Antibodies used	The following antibodies were used in this study;<br><br>pan-SOD1 (GTX100554) was purchased from Genetex (California, USA).<br>Misfolded SOD1 specific antibody (B8H10) was obtained from MediMabs (Montreal, Canada).<br>Actin (sc-1616), GST (sc-138), GFP (Green fluorescent protein; sc-8036) were purchased from Santa Cruz biotechnology (Santa Cruz, CA, USA).<br>TDP-43 antibody (10782-2-AP) was obtained from Proteintech (Rosemont, IL, USA).<br>Anti-FLAG (Sigma; F3165) was provided by Sigma Aldrich (St, Louis, Mo, USA).<br>HRP-conjugated goat anti-mouse, goat anti-rabbit and mouse anti-goat antibodies (Pierce, Thermo Fisher Scientific, Inc., Rockford, IL, USA) were used as secondary antibodies.
Validation	All antibodies used were validated by the respective commercial source for the application used in this manuscript

Eukaryotic cell lines

Policy information about cell lines

Cell line source(s)	HEK293 cells (ATCC, American Type Culture Collection).<br>SK-N-SH, SK-N-MC (KCLB, Korean Cell Line Bank).<br>Human fibroblast cell (9-year-old female, Coriell Cell Repositories)

Authentication	None of the cell lines have been authenticated.
Mycoplasma contamination	Cell lines were not tested for mycoplasma contamination but no indication of contamination was observed.
Commonly misidentified lines (See ICLAC register)	No commonly misidentified cell line were used.

Animals and other organisms

Policy information about studies involving animals; ARRIVE guidelines recommended for reporting animal research

Laboratory animals	All mice were maintained under temperature and light-controlled conditions (20~23 °C, 12 hr-12hr light/dark cycle) and provide autoclaved food and water.<br>Wild type and SOD1-G93A-Tg mice were used for in vivo experiment. B6SJL-Tg (SOD1G93A)1Gur/J mice were obtained from Jackson Laboratory (Stock No: 002726). Wild type mice indicate negative littermates of SOD1G93A-Tg mice
Wild animals	No wild animals were used in this study
Field-collected samples	No field-collected samples were used in this study
Ethics oversight	The experiments were performed in the Association for Assessment and Accerditation of Laboratory Animal Care certified facility, in compliance with animal policies approved by Korea National Institutes of Health Guide for the Care and Use of Laboratory Animals.

Note that full information on the approval of the study protocol must also be provided in the manuscript.
